# Use of a Hybrid Technique in the Surgical Correction of Severe Neuromuscular Scoliosis (Curves ≥85 Degrees): A Retrospective Study and Review of the Literature

**DOI:** 10.7759/cureus.86497

**Published:** 2025-06-21

**Authors:** Donald C Hefelfinger, Joseph D Henningsen, Sierra L Lindsey, Scott W Huff, Kelly Spiller, Arjun Minhas, Andrew W Froehle, Michael C Albert

**Affiliations:** 1 Department of Orthopaedic Surgery, Corewell Health East William Beaumont University Hospital, Royal Oak, USA; 2 Department of Orthopaedic Surgery, Wright State University, Boonshoft School of Medicine, Dayton, USA; 3 Department of Orthopaedic Surgery, MedStar Union Memorial Hospital, Baltimore, USA; 4 Department of Plastic Surgery, University of Cincinnati College of Medicine, Cincinnati, USA; 5 Department of Kinesiology and Health, Wright State University, Boonshoft School of Medicine, Dayton, USA; 6 Department of Orthopaedics, Dayton Children's Hospital, Dayton, USA

**Keywords:** hybrid fixation, neuromuscular scoliosis, pedicle screw, severe neuromuscular scoliosis, spinal fusion, sublaminar band

## Abstract

Background

Patients with neuromuscular scoliosis pose a challenge to surgeons due to rapid progression, diffuse vertebral involvement, osteopenia, and dysplasia. Surgical treatment of curves >50° results in high complication rates, and there remains no consensus on fixation techniques. Hybrid fixation utilizing pedicle screws and polyester sublaminar bands have been successful for surgical correction, but there is minimal data for major curves ≥85°. It’s important to investigate surgical techniques in this understudied, complex patient population. This study aims to report the use and efficacy of this hybrid construct to correct curves ≥85°.

Methods

This is a retrospective review of pediatric neuromuscular scoliosis cases performed by three surgeons at a single institution between 2009 and 2021. Patient inclusion criteria included Cobb angle ≥85°, no previous spine surgery, and a minimum of two years of follow-up. Demographics, including comorbidities, Gross Motor Function Classification System (GMFCS) and American Society of Anesthesiologists (ASA) physical status scores, were noted preoperatively. Preoperative and postoperative angles were measured and compared using paired t-tests. Maintenance of correction was evaluated at one year and the final follow-up visit. Complications were reported for comparison with other surgical techniques.

Results

Twenty patients were included in the study with an average follow-up of 4.5 years. Preoperative major curves improved from an average of 100.3° to 34.1° immediately after the surgery (P<0.001). Curve correction was maintained from immediate post-op to the final follow-up in all cases. Pelvic obliquity improved from an average of 29.7° to 8.3° (P<0.001). The post-op major complication rate was 30%, requiring an additional surgery or prolonged ICU stay.

Conclusions

For the most severe neuromuscular scoliosis patients, the hybrid construct technique improved and maintained Cobb angles and pelvic obliquity correction at a minimum follow-up of two years. The complication rates found in this complex patient population are comparable to or less than those reported after surgical correction of lesser curves. The hybrid construct proposed maintains correction in even the most severe major curves (≥85°), making it a viable option in this complex patient population.

## Introduction

Neuromuscular scoliosis (NMS), unlike adolescent idiopathic scoliosis (AIS), is rapidly progressive and often fails nonoperative management. Notably, the spinal curves observed in patients with NMS have increased variability compared to AIS, and often involve severe kyphosis [1-3]. Left untreated, NMS curve progression often leads to an inability to maintain seating posture, cardiopulmonary compromise, and imbalance of the head and neck [2-4]. Consequently, spinal fusion for patients with NMS is the definitive option to minimize symptoms, maximize quality of life, and improve the ability to care for patients [1,5,6]. Patients with NMS also tend to have higher rates of perioperative complications, often attributed to a higher burden of comorbidities, as well as comparatively poor nutrition, bone integrity, mobility, and personal hygiene [2,4,7-11]. 

Several studies report long-term outcomes (>two years) after surgical NMS curve correction using a variety of techniques. These studies have collectively shown an overall mean major curve correction of 61.4% where mean whole group preoperative Cobb angles ranged from 65° to 84° [3,12,13]. Techniques include Luque wires, pedicle screws, and more novel hybrid constructs utilizing a combination of polyester sublaminar bands (SLB) and pedicle screws [6,7,13]. The SLBs avoid risks associated with the other options, including screw pullout or wires that may cut through the lamina, while also attaining similar degrees of correction to sublaminar wire and all pedicle screw constructs. Additionally, the rate of proximal junctional kyphosis (PJK) with SLB has been reported at 5.8%, compared to 26% in patients with NMS undergoing posterior spinal fusion (PSF) [14]. Furthermore, a retrospective study evaluating SLB use in 378 patients reported a neurologic complication rate of 0.8%, which is comparable to neurologic complication rates of all other PSF approaches [15]. An additional retrospective cohort study looked at outcomes of 29 patients with NMS treated with the hybrid construct using SLB and pedicle screws with an average follow-up of 29 months [16]. This study reported an overall complication rate of 24% which was comparable to previous studies looking at sublaminar wire fixation and all pedicle screw fixation [16]. 

Thus, the hybrid construct appears to have unique advantages without compromising curve correction in patients with NMS, but the construct has yet to be evaluated in the treatment of more severe scoliosis curvature. The purpose of this paper was to report on the outcomes of correction using the hybrid SLB construct in severe NMS curves (≥85°). This retrospective study is one of few papers in literature to evaluate the correction of severe NMS scoliosis curves and does so while reporting on the efficacy and safety of a new construct in comparison to other PSF techniques.

## Materials and methods

All study procedures were approved by the Dayton Children's Hospital Institutional Review Board (IRB) prior to data collection. A retrospective chart review evaluated records of 49 patients with NMS seen by three surgeons at one institution from 2009 to 2021. Patients were included if they had an NMS diagnosis; spinal deformity equaling or exceeding 85° in the sagittal plane, frontal plane, or both; no previous spine surgery; and a minimum follow-up of two years. Of those initial 49 patients, only 20 met all the inclusion criteria.

Data on the participants' preoperative characteristics were collected, including primary diagnosis (cerebral palsy, Rett syndrome, muscular dystrophy, or other neuromuscular disorders including spinal muscular atrophy), age, body mass, gender, race, ambulatory status, indices of health status and motor function (American Society of Anesthesiologists Physical Status Classification System or ASA score, Gross Motor Function Classification System or GMFCS), preoperative tracheostomy and/or gastrostomy, and presence of individual comorbidities (gastroesophageal reflux disease or GERD, dislocated hip/skeletal comorbidity, neuromuscular, cardiac, nutritional, neurological, urinary, infection, or other comorbidities, and past history of seizures, pneumonia, or pulmonary compromise). Operative data included pediatric ICU (PICU) length of stay (LOS), total hospital LOS, operative time, number of spinal levels fused, number of pedicle screws used, number of SLBs used, cell saver volume, intraoperative blood loss, type of pelvic fixation (if applicable), and type of bone graft (if applicable). 

Primary outcomes were changes in radiographic parameters of spinal curvature, including major curve Cobb angle, maximal thoracic kyphosis, and upright pelvic obliquity. Other radiographic measurements included minor curve Cobb angle, lumbar lordosis, and sagittal vertical alignment. Radiographic measurements were collected by individuals trained across all measurements, reviewed by orthopedic residents, and validated through random spot checks by a fellowship-trained pediatric spine surgeon. Measurements were taken preoperatively, postoperatively, at one-year follow-up, and final follow-up. Secondary outcomes included major and minor complications. These were scored per Clavien-Dindo (CD) classification, defined similarly to studies of similar scope, with major complications requiring reoperation, extended inpatient management or permanent deficit, and minor complications requiring outpatient management or deviation from routine without additional operative intervention [17]. 

*Statistical Analysis* 

All statistical analysis was performed in SAS 9.4 (SAS Inc., Cary, NC), with significance set to α=0.05. Descriptive statistics were derived for all variables, including mean ± standard deviation (SD) for continuous variables (e.g. age), median and interquartile range (IQR) for counts (e.g., number of pedicle screws used), and frequencies for categorical variables (e.g. gender). To test the hypothesis that radiographic parameters decreased significantly between study time points (preoperative, immediate postoperative, one-year follow-up, and final follow-up), we used repeated measures linear mixed models to test for the main effect of time on each radiographic outcome. Post hoc pairwise comparisons were made for each outcome, comparing each follow-up time point vs. each prior time point, and adjusting for multiple comparisons (Tukey Honestly Significant Difference or HSD). Common language effect size (CLES) values were derived for each statistically significant difference between consecutive pairs of measurements [18]. Following our hypothesis that curvature values would decrease, this effect size measure gives the probability that individual patients have lower values at the later visit compared to the earlier visit. 

## Results

A total of 20 patients met the study's inclusion criteria, the most common cause of exclusion being curvature <85°. Among the included patients, the primary diagnoses were cerebral palsy (n=13), Rett syndrome (n=2), Duchenne muscular dystrophy (n=1), and other diagnoses (n=4). Mean final follow-up length was 4.5±2.2 years (range: 2.0-11.5 years). Just 20% of patients were ambulatory preoperatively, vs. 25% postoperatively (one patient improved from non-ambulatory to ambulatory). Other preoperative subject characteristics, including comorbidities, are presented in Table 1.

**Table 1:** Preoperative patient characteristics (n=20) GMFCS: Gross Motor Function Classification System; ASA: American Society of Anesthesiologists. General patient characteristics, functional status, and other preoperative comorbidities were included. ^a^Reported statistics are mean±standard deviation for continuous variables, median (interquartile range) for counts, and frequencies (%) for categorical variables.

Variables	Descriptive statistics^a^
Age (years)	13.8±2.7
Body mass (kg)	35.5±9.0
Gender (%)	
Male	0.5 (50%)
Female	0.5 (50%)
Race (%)	
White	0.95 (95%)
Black	0.05 (5%)
GMFCS (%)	
I/II	0.05 (5%)
III	0.10 (10%)
IV/V	0.85 (85%)
ASA score	2.9±0.9
Comorbidities	
Tracheostomy	0.10 (10%)
Gastrostomy	0.65 (65%)
Pneumonia	0.25 (25%)
Pulmonary compromise	0.50 (50%)
Seizures	0.80 (80%)
GERD	0.65 (65%)
Dislocated hip/skeletal	0.35 (35%)
Neuromuscular	1.00 (100%)
Cardiac	0.25 (25%)
Nutritional	0.45 (45%)
Neurological	0.65 (65%)
Urinary	0.35 (35%)
Infection	0.10 (10%)
Other	0.40 (40%)
Total (interquartile range)	4 (2-5)

Operative details are presented in Table 2. 

**Table 2:** Operative details IQR: Interquartile; LOS: Length of stay. Details regarding the patient’s operation included the type of pelvic fixation used, the use of bone graft, as well as the implants that were used. Details on the patient’s hospital course included time spent in the ICU and total LOS. ^a^Reported statistics are mean±SD for continuous variables, median (IQR) for counts, and frequencies (%) for categorical variables.

Variables	Descriptive statistics^a^
PICU LOS (days)	3.2±2.7
Total LOS (days)	10.7±7.5
Operative time (min)	357.5±94.5
No. levels fused (IQR)	17 (16.75-17)
No. screws (IQR)	17 (14.75-20.25)
No. bands (IQR)	10 (9-12)
Cell saver volume (L)	0.5±0.3
Intraoperative blood loss (L)	1.2±0.6
Pelvic fixation (%)	
None	0.30 (30%)
Galveston	0.35 (35%)
Sacral alar iliac	0.35 (35%)
Bone graft (%)	
None	0 (0%)
Allograft	0.15 (15%)
Mixture	0.85 (85%)

**Figure 1:**
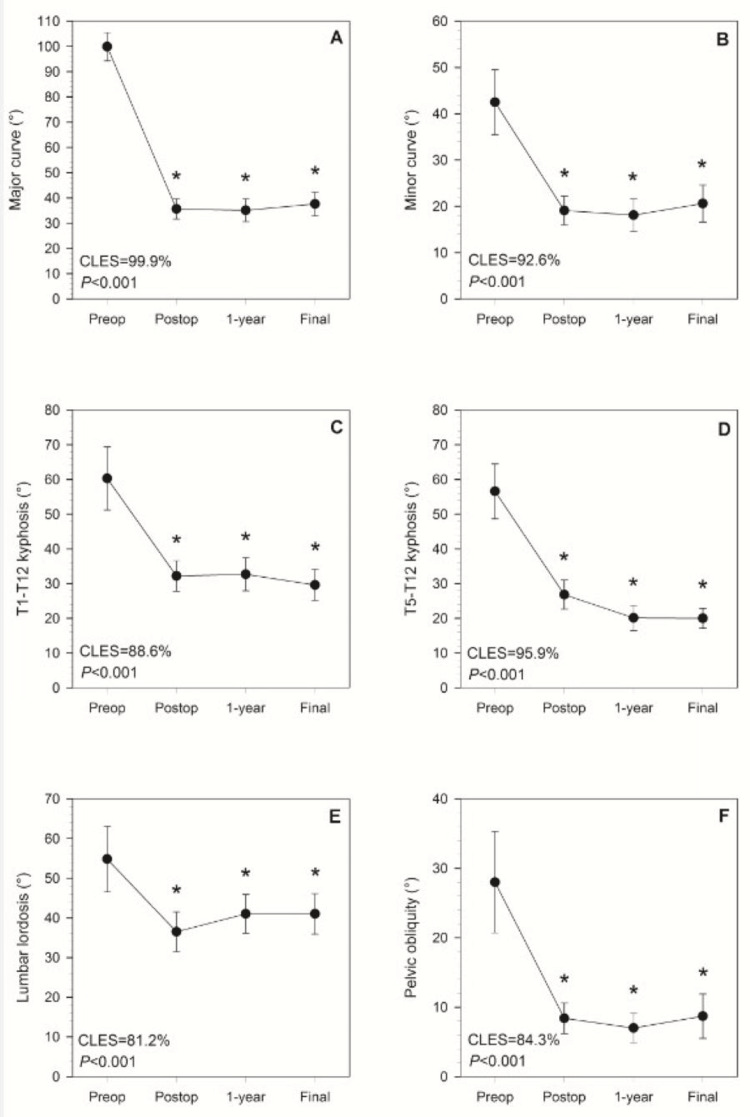
Time-series plots for different radiographic measurements at preoperative, immediate postoperative, one-year follow-up, and final follow-up time points Time-series plots showing sample means and 95% confidence intervals of the mean at preoperative, immediate postoperative, one-year follow-up, and final follow-up time points for (A) major curve, (B) minor curve, (C) T1-T12 kyphosis, (D) T5-T12 kyphosis, (E) lumbar lordosis, and (F) pelvic obliquity. Reported P-values are for the overall main effect of time, and asterisks indicate that the mean value at a given time point differs significantly from preoperative baseline (for each, P<0.001). There were no differences in values between any pair of postoperative time points for any variable (for each, P≥0.077). The reported percentages are common language effect size (CLES), which gives the probability that an individual patient would have a lower value immediately postoperatively compared to preoperative baseline.

With the exception of sagittal vertical alignment (P=0.678), the main effect of time was significant for each radiographic spinal curvature variable (for each, P<0.001) (Figure 1).

**Figure 2:**
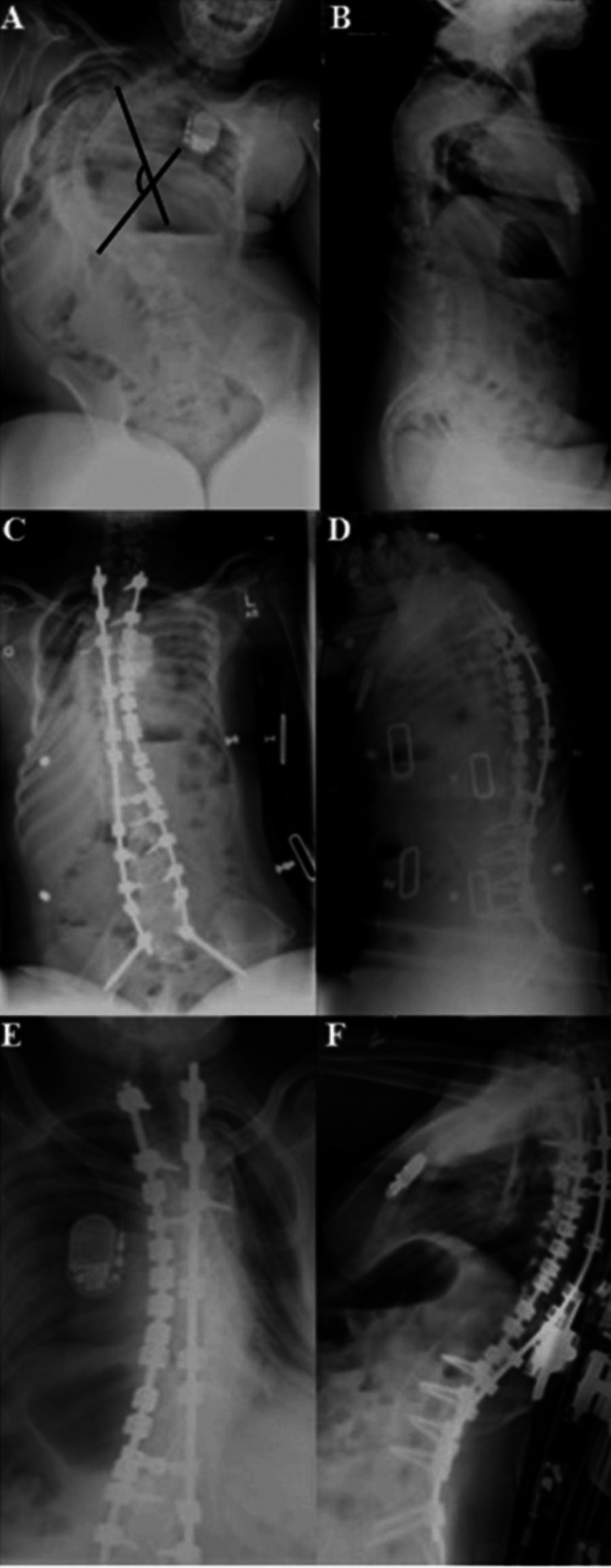
Preoperative, immediate postoperative, and follow-up radiographs of a patient with neuromuscular scoliosis with a major Cobb angle of 136° Anteroposterior (AP) and lateral radiographs of a 12-year-old female patient with neuromuscular scoliosis with a major Cobb angle of 136° (black lines). (A and B) Preoperative images; (C and D) Immediate postoperative images, major Cobb angle corrected to 40°; (E and F) Images at final follow-up visit, 3.5 years post-operatively, with a major Cobb angle of 54°. This patient underwent surgical spine fixation using the hybrid technique. Images demonstrate maintenance of major angle correction at her final follow-up visit.

Preoperative major curves ranged from 85°-136° (100.3°±15.3°) with immediate postoperative major curves ranging from 19°-57° (34.1°±11.0°). In each case, the post hoc analysis showed that curvatures decreased significantly from preoperative to immediate postoperative measurements (for each, P<0.001), and that subsequent follow-up measurements were also significantly lower than preoperative values (for each, P<0.001) (Figure 2).

There were no statistically significant differences between values at postoperative visits for any variable (for each, P≥0.077). CLES for the preoperative-immediate postoperative decreases were large, ranging between 84.6% and 99.9%. 

All but one patient experienced at least one surgical complication, with 15% falling into Grade I, 20% in Grade II, 30% in Grade III, 35% in Grade IV, and 0% in Grade V (CD classification). All minor complications were treated successfully without surgery, including transient foot drop, postoperative ileus requiring nasogastric tube, surgical site infection treated with antibiotics, and pleural effusion. One patient (5%) returned to surgery due to implant failure and four patients (20%) returned to surgery due to deep surgical site infection or wound dehiscence requiring washout (two for deep wound infections and two for superficial wound dehiscence that could not be treated with antibiotics alone and required surgical washout). One patient required prolonged PICU stay due to respiratory failure postoperatively. Table 3 presents rates of all surgical complications in this sample.

**Table 3:** Surgical or postoperative complications PIC: Peripherally inserted catheter; Details regarding the percentage of surgical and postoperative complications in this patient group. %Y refers to the percentage of patients for whom the data entry for that variable was "yes", i.e. they experienced the complication. Major complication rate (requiring subsequent surgery or prolonged ICU stay) was 0.30 (30%).

Complication	Rate
Implant failure requiring second surgery (%Y)	0.05 (5%)
Pseudarthrosis (%Y)	0 (0%)
Transient foot drop (%Y)	0.05 (5%)
Gastrointestinal (%Y)	0.45 (45%)
Pulmonary (%Y)	0.40 (40%)
PIC line thrombosis (%Y)	0.15 (15%)
PIC line infection (%Y)	0.10 (10%)
Pressure sores (%Y)	0 (0%)
Wound dehiscence managed nonoperatively (%Y)	0.15 (15%)
Superficial surgical site infection (SSI), antibiotics only (%Y)	0.10 (10%)
Deep SSI/Wound dehiscence requiring second surgery (%Y)	0.20 (20%)
Urinary tract infection (%Y)	0.05 (5%)

Two patients ultimately died during their follow-up. These deaths occurred after the minimum two-year follow-up period and were likely a result of other severe medical comorbidities and not a result of their surgical intervention. Patients with normal bloodwork (albumin and prealbumin levels) had a median number of complications of 3.5, with an IQR of 2.0-5.0. Patients with abnormal bloodwork had a median number of complications of 4.0, with an IQR of 3.0-5.0. When a Wilcoxon rank-sum test was used, this difference was not statistically significant (P=0.708). 

## Discussion

The current standard of care for the surgical management of NMS is aimed at stabilizing the spinal curvature, preventing further progression, balancing the pelvis, and improving pulmonary function. While many constructs are able to maintain NMS curvature correction long-term, they are fraught with high complication rates. Notable complications include hardware failure (especially in the Luque wire technique), high infection rates (5.5% compared to 1.4% in patients with AIS), cardiopulmonary complications, non-union, skin breakdown, and even death. 

Hybrid fixation with polyester SLBs and pedicle screws has been proposed as an optimal surgical method for patients with NMS due to their associated nutritional deficiencies and osteopenia that puts them at a high risk for screw pullout and wire cutout. Furthermore, osteopenia and vertebral dysplasia can make pedicle screw insertion at each level for rod fixation technically difficult for surgeons. Hybrid fixation has demonstrated comparable curve correction and decreased rates of PJK, while minimizing the use of pedicle screws. A prior investigation of hybrid fixation constructs in 29 patients with NMS with an average preoperative major Cobb angle of 71° demonstrated favorable results, where 97% of patients achieved appropriate postoperative sagittal alignment, with only 2% demonstrating a loss of sagittal correction at final follow-up. Coronal realignment was similarly robust, with an average coronal correction of 69% that was not lost in any patients [16]. 

This paper is the first to show the effectiveness and safety of hybrid fixation even in the most severe NMS curves. To our knowledge, no paper has presented a cohort of this size with all major Cobb angles >85° treated with posterior fixation alone. The average preoperative Cobb angle of the cohort discussed is 100°, which is 16° and 29° greater than prior investigations of similar nature [13,16]. Our results show that a hybrid construct allows for an average of 64.6% and 49.4% correction for major and minor curves, respectively, which is maintained at an average follow-up of 4.5 years. These rates are comparable to or better than literature reported for less severe curves. Notably, many of these correction percentages are higher than those reported in literature, as growing rods show an average of 50% correction, 54% for the Luque-Galveston correction, and 61% for the fusionless technique. Our major complication rate of 30% (one implant failure requiring second surgery, four deep surgical site infections/wound dehiscence requiring second surgery, and one respiratory failure requiring prolonged PICU stay) is also comparable to that reported in literature for less severe curves. 

Limitations of this study are common to all retrospective studies on rare pathologies. Our small sample size limits the discussion of complication rates; however, curves of this magnitude are rare, which makes the study of this pathology difficult. Ideally, patients should undergo operative correction of NMS deformities at less severe degrees of curvature, but several intrinsic and extrinsic factors influence the timing of appropriate surgical care. Previous studies have elucidated multiple socioeconomic and demographic factors related to delayed presentation in patients with AIS [19-22]. Distrust of the healthcare system, poor understanding of the condition, limited screening opportunities, and concerns regarding surgery are extrinsic parental factors that have been shown to influence late presentation in patients of AIS and may apply to parents of children with NMS [19-22]. Anecdotally, patients within our cohort were lost to surgeon follow-up for prolonged periods of time prior to undergoing operative intervention, with several of the aforementioned factors likely conferring some degree of influence. The operations were performed by three surgeons at a high-volume Level 1 children’s hospital which may limit the external validity of our results to lower volume centers with less experience. Without a control group, comparison of the hybrid technique can only be made to available literature. This is also limited as much of the literature examines curves below 70°. More research on surgical intervention for severe NMS is needed to optimize surgical techniques and enhance all facets of care from preoperative optimization to postoperative management. 

## Conclusions

This retrospective study illustrates the effectiveness and safety of polyester SLBs with pedicle screw fixation for the management of severe NMS. Despite the most severe curves in a complex patient population, hybrid fixation achieved and maintained curve correction with complication rates comparable to published literature. Future studies are needed to optimize preoperative care, intraoperative technique and minimize post-operative complications in this complex patient population. 

## References

[1] Tsirikos AI, Chang WN, Dabney KW, Miller F, Glutting J (2003). Life expectancy in pediatric patients with cerebral palsy and neuromuscular scoliosis who underwent spinal fusion. Dev Med Child Neurol.

[2] Brooks JT, Yaszay B, Bartley CE (2019). Do all patients with cerebral palsy require postoperative intensive care admission after spinal fusion?. Spine Deform.

[3] Toovey R, Harvey A, Johnson M, Baker L, Williams K (2017). Outcomes after scoliosis surgery for children with cerebral palsy: a systematic review. Dev Med Child Neurol.

[4] Toll BJ, Samdani AF, Janjua MB, Gandhi S, Pahys JM, Hwang SW (2018). Perioperative complications and risk factors in neuromuscular scoliosis surgery. J Neurosurg Pediatr.

[5] Tsirikos AI, Mains E (2012). Surgical correction of spinal deformity in patients with cerebral palsy using pedicle screw instrumentation. J Spinal Disord Tech.

[6] Fuhrhop SK, Keeler KA, Oto M (2013). Surgical treatment of scoliosis in non-ambulatory spastic quadriplegic cerebral palsy patients: a matched cohort comparison of unit rod technique and all-pedicle screw constructs. Spine Deform.

[7] Nectoux E, Giacomelli MC, Karger C, Herbaux B, Clavert JM (2010). Complications of the Luque-Galveston scoliosis correction technique in paediatric cerebral palsy. Orthop Traumatol Surg Res.

[8] Luhmann SJ, Furdock R (2019). Preoperative variables associated with respiratory complications after pediatric neuromuscular spine deformity surgery. Spine Deform.

[9] Adams AJ, Refakis CA, Flynn JM (2019). Surgeon and caregiver agreement on the goals and indications for scoliosis surgery in children with cerebral palsy. Spine Deform.

[10] Samdani AF, Belin EJ, Bennett JT (2016). Major perioperative complications after spine surgery in patients with cerebral palsy: assessment of risk factors. Eur Spine J.

[11] Nishnianidze T, Bayhan IA, Abousamra O, Sees J, Rogers KJ, Dabney KW, Miller F (2016). Factors predicting postoperative complications following spinal fusions in children with cerebral palsy scoliosis. Eur Spine J.

[12] Sitoula P, Holmes L Jr, Sees J, Rogers K, Dabney K, Miller F (2016). The long-term outcome of early spine fusion for scoliosis in children with cerebral palsy. Clin Spine Surg.

[13] Beckmann K, Lange T, Gosheger G (2016). Surgical correction of scoliosis in patients with severe cerebral palsy. Eur Spine J.

[14] Battista C, Wild C, Kreul S, Albert M (2018). Prevention of proximal junctional kyphosis & failure using sublaminar bands in a hybrid construct in pediatric kyphosis deformity. Int J Spine Surg.

[15] Desai SK, Sayama C, Vener D, Brayton A, Briceño V, Luerssen TG, Jea A (2015). The feasibility and safety of using sublaminar polyester bands in hybrid spinal constructs in children and transitional adults for neuromuscular scoliosis. J Neurosurg Pediatr.

[16] Albert MC, LaFleur BC (2015). Hybrid fixation with sublaminar polyester bands in the treatment of neuromuscular scoliosis: a comparative analysis. J Pediatr Orthop.

[17] Clavien PA, Barkun J, de Oliveira ML (2009). The Clavien-Dindo classification of surgical complications: five-year experience. Ann Surg.

[18] Lakens D (2023). Calculating and reporting effect sizes to facilitate cumulative science: a practical primer for t-tests and ANOVAs. Front Psychol.

[19] Erkkila IP, Reynolds CA, Weissman JP, Levine OP, Aronson H, Knoll JM, Larson JE (2023). Factors associated with presentation of severe adolescent idiopathic scoliosis. J Pediatr Orthop Soc North Am.

[20] Lee JZ, Lam DJ, Lim KB (2014). Late presentation in adolescent idiopathic scoliosis: who, why, and how often?. J Pediatr Orthop B.

[21] Motyer G, Dooley B, Kiely P, Fitzgerald A (2021). Parents' information needs, treatment concerns, and psychological well-being when their child is diagnosed with adolescent idiopathic scoliosis: a systematic review. Patient Educ Couns.

[22] Willson LR, Rogers LG, Gingrich N, Shearer K, Hryniuk SS (2021). Meeting the needs of parents of children with scoliosis: a qualitative descriptive study. Glob Qual Nurs Res.

